# Restoration of rhythmicity in diffusively coupled dynamical networks

**DOI:** 10.1038/ncomms8709

**Published:** 2015-07-15

**Authors:** Wei Zou, D. V. Senthilkumar, Raphael Nagao, István Z. Kiss, Yang Tang, Aneta Koseska, Jinqiao Duan, Jürgen Kurths

**Affiliations:** 1 School of Mathematics and Statistics, Huazhong University of Science and Technology, Wuhan 430074, China.; 2 Center for Mathematical Sciences, Huazhong University of Science and Technology, Wuhan 430074, China.; 3 Potsdam Institute for Climate Impact Research, Telegraphenberg, D-14415 Potsdam, Germany.; 4 Centre for Nonlinear Science and Engineering, School of Electrical and Electronics Engineering, SASTRA University, Thanjavur 613401, India.; 5 Department of Chemistry, Saint Louis University, 3501 Laclede Avenue, St Louis, Missouri 63103, USA.; 6 The Key Laboratory of Advanced Control and Optimization for Chemical Processes, Ministry of Education, East China University of Science and Technology, Shanghai 200237, China.; 7 Department of Systemic Cell Biology, Max Planck Institute of Molecular Physiology, Dortmund D-44227, Germany.; 8 Research Centre for Computer Science and Information Technologies, Macedonian Academy of Sciences and Arts, Skopje, Macedonia.; 9 Department of Applied Mathematics, Illinois Institute of Technology, Chicago, Illinois 60616, USA.; 10 Institute of Physics, Humboldt University Berlin, D-12489 Berlin, Germany.; 11 Institute for Complex Systems and Mathematical Biology, University of Aberdeen, Aberdeen AB24 3FX, UK.; 12 Department of Control Theory, Nizhny Novgorod State University, Gagarin Avenue 23, 606950 Nizhny Novgorod, Russia.

## Abstract

Oscillatory behaviour is essential for proper functioning of various physical and biological processes. However, diffusive coupling is capable of suppressing intrinsic oscillations due to the manifestation of the phenomena of amplitude and oscillation deaths. Here we present a scheme to revoke these quenching states in diffusively coupled dynamical networks, and demonstrate the approach in experiments with an oscillatory chemical reaction. By introducing a simple feedback factor in the diffusive coupling, we show that the stable (in)homogeneous steady states can be effectively destabilized to restore dynamic behaviours of coupled systems. Even a feeble deviation from the normal diffusive coupling drastically shrinks the death regions in the parameter space. The generality of our method is corroborated in diverse non-linear systems of diffusively coupled paradigmatic models with various death scenarios. Our study provides a general framework to strengthen the robustness of dynamic activity in diffusively coupled dynamical networks.

## Abstract (web summary)

Oscillatory behaviour is essential for proper functioning of several processes, yet quenching phenomena can lead to steady states with suppressed oscillations. Here the authors present a scheme to revoke these states in diffusively coupled networks, and demonstrate their approach on a chemical oscillator.

## Graphical abstract

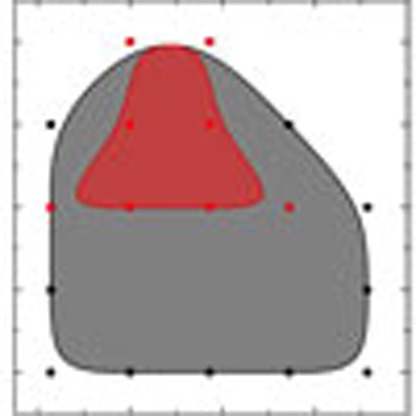

Modelling coupled oscillators offer an alternative and effective approach aimed at understanding a plethora of intriguing self-organizing phenomena in diverse fields of non-linear sciences ranging from physics, chemistry, biology and neuroscience to engineering^1^,^2^,^3^,^4^. The phenomenon of oscillation quenching (oscillation suppression) is a fascinating emerging behaviour, whereby individual oscillatory systems cease to oscillate when coupled, which results into the emergence of stationarity of the whole-coupled system^5^,^6^. One of the early observations of oscillation quenching dates back to the 19th century by Lord Rayleigh, who found that two organ pipes of the same pitch standing side by side mutually suppressed their vibration^2^. Later, this phenomenon was observed in chemical reactions^7^,^8^, lasers^9^, electronic circuits^10^, neural oscillators^11^ and so on. Models based on coupled non-linear oscillators have been extensively used to understand the intricacies of oscillation quenching for more than two decades^5^,^6^. Very recently, two structurally distinct oscillation quenching processes have been clearly distinguished as: (i) amplitude death (AD) and (ii) oscillation death (OD)^6^,^12^. Generally, AD refers to stabilization of an already existing homogeneous steady state (HSS) to which the coupled units are entrained. In contrast, OD is manifested as a novel inhomogeneous steady state (IHSS), which occurs due to symmetry breaking of the coupled systems, and the distinct units then populate different branches of the same IHSS^6^,^12^.

The phenomena of AD and OD can be responsible for a loss of intrinsic dynamics, which may lead to a large degree of degradation in the dynamic performance of physical systems. For example, the onset of AD and OD could seriously weaken and even completely destroy the transmission of electricity in a power grid, where the energy flows along transmission lines arise from periodic oscillations of the rotational phase of generators at the frequency of 50 Hz (60 Hz)^13^,^14^,^15^. On the other hand, sustained oscillations represent one of the most striking and ubiquitous manifestations of dynamic evolution in distinct real-world systems^16^,^17^, such as spiking neural networks, cardiac and respiratory systems, and El Niño/Southern Oscillation in Earth's ocean and atmosphere and so on. Moreover, rhythmic behaviours play an essential function at all levels of physiological processes, which make it possible to organize and control the activity of populations of cells^18^. For instance, it has been proposed that highly synchronized theta frequency oscillations serve as a temporal organizer for cortex in the hippocampus^19^. These examples furnish the robustness of dynamic activity as a main prerequisite for proper functioning of multitude natural and man-made networks. Thus, oscillation quenching in such real systems is destructive and fatal to robustness of rhythmic behaviours, which is undesirable and should be circumvented.

For a couple of decades, the major research focus was centred at identifying various scenarios that facilitate the emergence of AD and OD in coupled oscillatory systems^20^,^21^,^22^,^23^,^24^,^25^,^26^,^27^,^28^,^29^,^30^,^31^,^32^,^33^,^34^. Only in the recent past, a significant attention has been devoted to explore possibilities of restoring rhythmic activity after the dynamic activity of the coupled networks irreversibly fails, as a means to understand sustained oscillatory mechanisms of natural systems^35^,^36^,^37^,^38^,^39^. However, a general technique to efficiently overcome the phenomenon of oscillation quenching in coupled oscillatory systems remains as an open challenge as highlighted in a recent review^5^.

In this paper, we propose a rather simple and efficient approach for revoking oscillation suppression: a limiting factor in the diffusive coupling effectively recovers rhythmic behaviour of coupled networks, whose dynamic activity has been destroyed by the diffusive interaction of the elements. We demonstrate that this simple limiting factor can destabilize the stable HSS and IHSS to wipe out the onset of both AD and OD in diffusively coupled paradigmatic non-linear oscillators. Astonishingly, even a minute deviation from the normal diffusive coupling can reduce drastically the stable regimes of AD and OD in the parameter space. Both the scheme and the phenomenon are clearly verified in experiments using electrochemical oscillators: the stability of the HSS of current generating chemical reactions emanating from dissolution of nickel wires is revoked by a simple adjustment of the nature of the interactions. The proposed method is highly efficient and quite robust in regaining sustained oscillations in networks of diffusively coupled non-linear oscillators, where the dynamic activity has been damaged and lost as a consequence of the diffusive interaction. Our study leads to a significant improvement of our understanding of the role of diffusive coupling in controlling oscillatory activity of coupled complex non-linear systems.

## Results

### Two coupled Stuart–Landau oscillators

In the following, we will demonstrate our approach using the paradigmatic model of coupled Stuart–Landau oscillators, describing dynamics near a supercritical Hopf bifurcation (further examples of oscillatory systems are discussed in the Supplementary Notes 1–5). Systems of coupled Stuart–Landau oscillators have served as an ideal model for exploring the mechanisms of AD by analytical means for more than a couple of decades^20^,^21^,^22^,^23^,^24^,^25^,^26^,^27^,^28^. So far, numerous scenarios such as frequency mismatch^20^,^21^, time-delayed coupling^22^,^23^,^24^,^25^,^26^, and conjugate and dynamic couplings^27^,^28^ and so on have been reported to induce AD in coupled Stuart–Landau oscillators. On the other hand, a burst of recent research activity has revealed that OD can even occur in coupled identical Stuart–Landau oscillators when the coupling destroys the rotational symmetry of the system^12^,^31^,^32^,^33^,^34^. Here we will establish that the onset of both AD and OD under all the above-mentioned scenarios can be effectively revoked to retain self-sustained oscillations by a very simple coupling scheme.

Let us begin with two coupled Stuart–Landau oscillators^1^


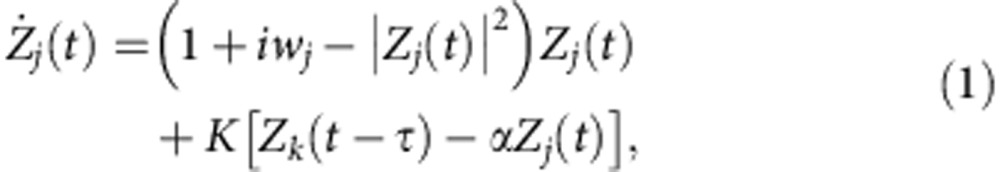


where *j*, *k*=1, 2 (*j*≠*k*), *Z*_*j*_=*x*_*j*_+*iy*_*j*_ and *w*_*j*_ are the complex amplitude and the natural frequency of the *j*th oscillator, respectively, *K* is the coupling strength, and *τ* is the propagation delay. In contrast to the studies on the traditional diffusive interaction, here we introduce a new factor *α* in the coupling. The new ingredient offers the opportunity to better characterize the diffusion process at the interface of a wide range of natural systems. In addition, the new feedback factor *α* acts as a control parameter: *α*=1 corresponds to a normal diffusive coupling, whereas *α*=0 refers to a direct coupling scheme. Thus, the new diffusive coupling may naturally occur in more realistic circumstances as a bridge-linking direct coupling and normal diffusive interaction. We will mainly probe its influence on the collective dynamics of coupled non-linear systems, and reveal that an extremely small detuning of *α* from the normal diffusion has a significant impact on the oscillatory activity of the system.

For zero-coupling strength (*K*=0), the *j*th Stuart–Landau oscillator exhibits a stable limit-cycle oscillation 
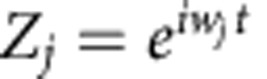
 and has an unstable focus at *Z*_*j*_=0. The occurrence of AD in the coupled system (Equation 1) for *α*=1 has been well studied in ref. 20 for *τ*=0 and in ref. 22 for *τ*>0. In this respect, Aronson *et al*.^20^ asserted that AD occurs for sufficiently disparate frequencies, whereas Reddy *et al*.^22^ showed that AD occurs even for coupled identical oscillators if *τ*>0. We next show that the coupling with *α*<1 overrides the quenching effects by revoking the stability of AD to regain oscillations in the respective AD parameter space.

When the coupled system (Equation 1) experiences AD, the HSS at the origin is stabilized. The condition for the onset of AD can be determined from a linear stability analysis around *Z*_1_=*Z*_2_=0. Assuming that the linear perturbations vary as *e*^*λt*^, the characteristic equation can be written as


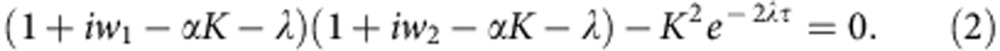


**Figure 1:**
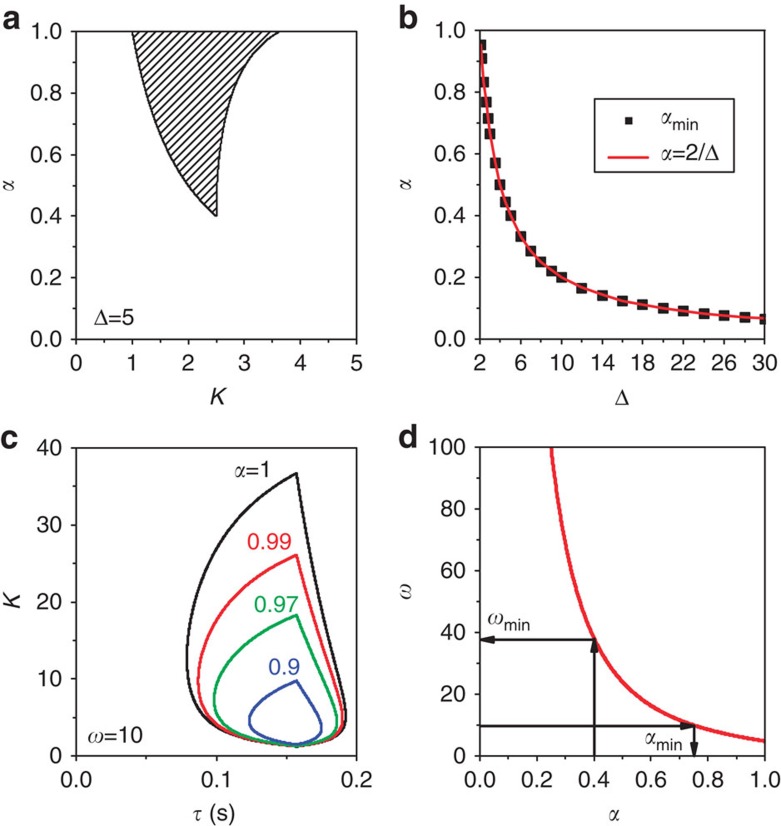
AD with frequency mismatch and discrete time delay. (**a**) The stable AD interval versus *α* with frequency mismatch Δ=|*w*_1_−*w*_2_|=5 and *τ*=0. AD is impossible for any coupling strength *K* with *α*<*α*_min_=0.4. (**b**) The critical *α*_min_ versus frequency mismatch Δ for *τ*=0. (**c**) AD islands in the parameter (*τ*, *K*) space for different values of *α* with *w*_1_=*w*_2_=*w*=10. (**d**) The minimal value *α*_min_ versus *w*. If *α*<*α*_min_ AD is completely revoked for all *τ* and *K*.

Thus, for *τ*=0, the stability condition of AD is: 1/*α*<*K*<*γ*(*α*), where *γ*(*α*)=(1+Δ^2^/4)/2 if *α*=1 and 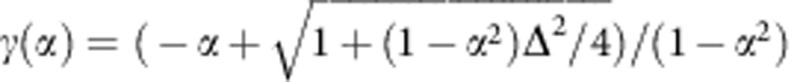 if *α*<1, where Δ=|*w*_1_−*w*_2_|. We find that the spread of the stable HSS (AD) in the (*K*, *α*) space narrows down on decreasing *α* for Δ>2 (Fig. 1a for Δ=5) and eventually vanishes if *α*<*α*_min_. The value of *α*_min_ can be analytically deduced as *α*_min_=2/Δ, and exactly agrees with the simulation results in Fig. 1a. The exact fit between the simulation *α*_min_ (black squares) and the analytical *α*_min_ (red line) is depicted in Fig. 1b as a function of *α* and Δ. Thus, it is clear that the feedback factor *α* plays a crucial role in determining the stability of HSS, which restores oscillations in stable HSS (AD) regimes by revoking its stability for *α*<*α*_min_ in coupled non-identical oscillators.

The characteristic equation (2) can be further simplified as


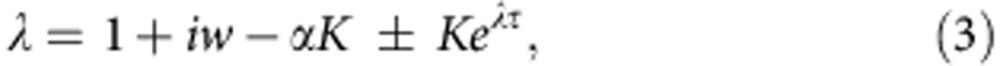


for *w*_1_=*w*_2_=*w*, whose roots can be analytically resolved as


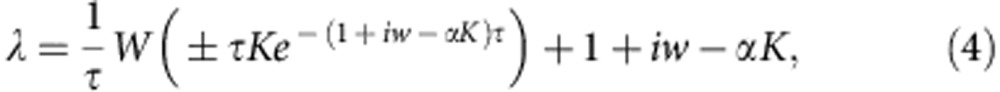


where *W* is the Lambert function defined as the inverse map of *G*(Λ)=Λ*e*^Λ^ (ref. 40). Moreover, the boundaries of stable HSS (AD islands) on the (*τ*, *K*) plane for *w*_1_=*w*_2_=*w* can be explicitly derived as


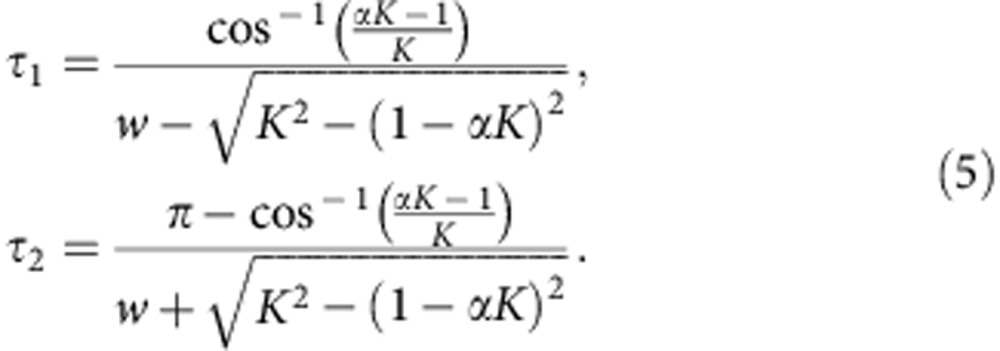


AD islands in the (*τ*, *K*) plane for *α*=1, 0.99, 0.97 and 0.9 are illustrated in Fig. 1c for *w*=10. Surprisingly, we observe that the AD island shrinks greatly as *α* is slightly decreased, and completely disappears if *α*<*α*_min_≈0.748. Now, the relation between *α*_min_ and *w* can be deduced as


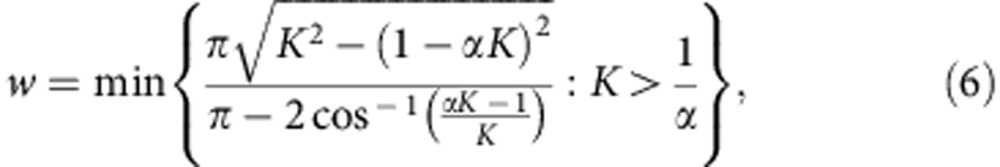


which is depicted in Fig. 1d using the red line. This line demarcates the unstable HSS on its left side for any combinations of (*τ*, *K*) and stable HSS on its right side for a certain set of (*τ*, *K*). A minimal *α*_min_ necessary for revoking the stability of HSS for a given *w* on the (*τ*, *K*) plane and vice versa can also be determined from the above relation. Hence, it is strongly confirmed that the feedback factor *α* successfully induces oscillations in stable HSS (AD) regimes of delay-coupled identical oscillators for *α*<*α*_min_.

**Figure 2:**
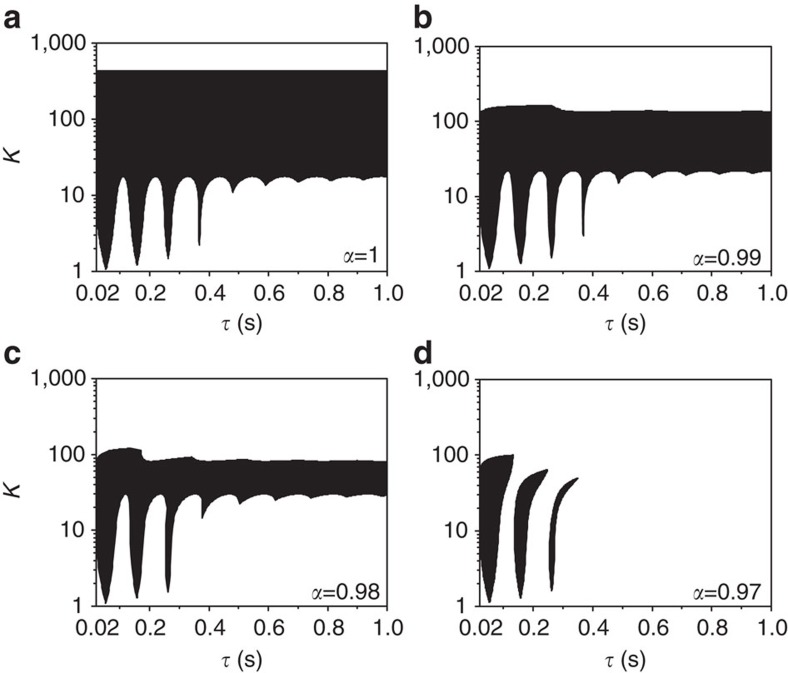
AD with distributed time delays. (**a**–**d**) The stability region of AD with time delays uniformly distributed over *τ*±0.02 for *α*=1, 0.99, 0.98 and 0.97, respectively. The stable AD region breaks into three disconnected and bounded islands for *α*=0.97. A feeble decrease in the value of the limiting factor *α* from unity drastically shrinks the stable regions of AD in the parameter space, which implies that dynamic activity is efficiently restored. AD is impossible for any values of *τ* and *K* if *α*<*α*_min_=0.424. The intrinsic frequencies are fixed as *w*_1_=*w*_2_=*w*=30.

Atay^25^ reported that distributed delays favour a stabilization of HSS in a larger parameter space compared with discrete delays. In what follows, we will explore the impact of the feedback factor *α* on the two coupled Stuart–Landau oscillators with distributed delays


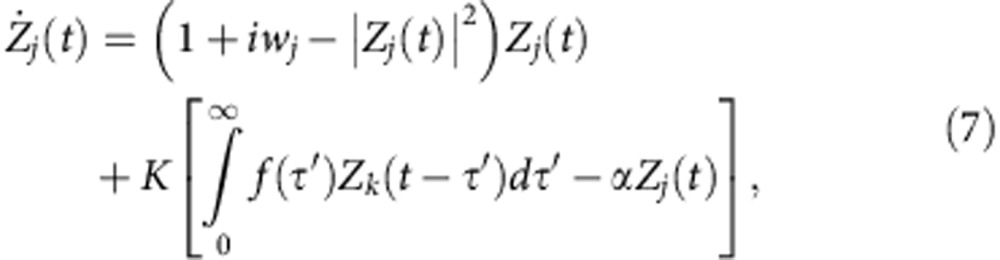


where *f* represents a distributed delay kernel. If *f* is the delta function *f*(*τ*′)=*δ*(*τ*′−*τ*), one can recover the coupled system (Equation 1). Here we will concentrate on *w*_1_=*w*_2_=*w*, and a uniformly distributed delay kernel *f*(*τ*′)=1/(2*β*) if |*τ*′−*τ*|<*β* and zero elsewhere as in ref. 25. The kernel *f* approaches *δ*(*τ*′−*τ*) as *β*→0. For *α*=1, Atay^25^ showed that the AD region becomes unbounded along *τ* when *β*>0.008 for *w*=30 as illustrated in Fig. 2a with *β*=0.02. To our surprise, on a very small decrease of *α* from 1, we find that the spread of the AD regime shrinks sharply as shown in Fig. 2b,c for *α*=0.99 and 0.98, respectively. Moreover, the stable AD region breaks into three disjoint and bounded islands for *α*=0.97 (Fig. 2d). Decreasing *α* further wipes off the AD region completely from the whole parameter space (*α*<*α*_min_=0.424), thereby restoring stable oscillations by switching the stability of stable HSS (AD). Hence, the feedback factor *α* circumvents AD in coupled oscillators even for time delays distributed over an interval, thereby corroborating the robust nature of *α* in invoking oscillations by revoking the stability of AD.

Conjugate and dynamic couplings are two forms of novel and distinct interactions facilitating AD in coupled identical oscillators without any propagation delays^27^,^28^. Could the presence of the feedback factor *α* be capable of eradicating the onset of AD in the coupled systems with both coupling configurations? To clarify it, we thus consider the following systems of Stuart–Landau oscillators with conjugate coupling


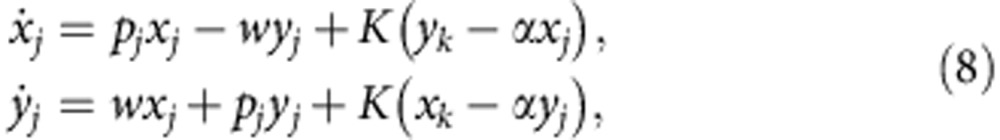


where *p*_*j*_=1−|*Z*_*j*_|^2^=1−*x*_*j*_^2^−*y*_*j*_^2^, *j*, *k*=1,2 (*j*≠*k*), and with dynamic coupling


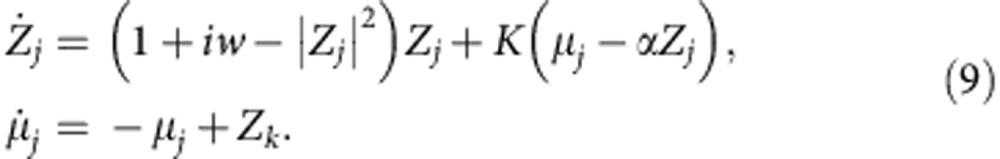


**Figure 3:**
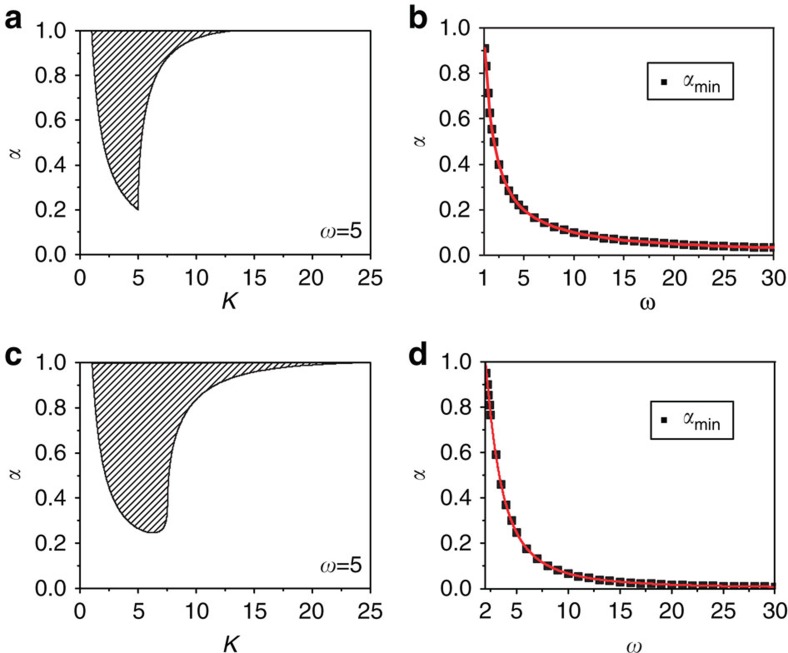
AD with conjugate and dynamic couplings. (**a**,**c**) The stable AD interval *K* versus *α* for conjugate and dynamic couplings, respectively. *w*=5 is fixed. (**b**,**d**) The minimal *α*_min_ versus *w* for conjugate and dynamic couplings, respectively. The black squares denote numerical results, and the red line represents theoretical predictions. The scheme for restoration of rhythmicity is valid for coupled systems with distinctly different interactions.

The underlying mechanisms of AD induced by conjugate and dynamic couplings are completely different compared to that of the previous cases. In conjugate coupling, the individual systems are coupled via dissimilar variables^27^, whereas additional variables are involved to describe the dynamic coupling^28^. For both coupling scenarios, AD can be identified by performing a linear stability analysis around the origin of the coupled systems (Equations 8 and 9). The corresponding characteristic equations can be obtained as


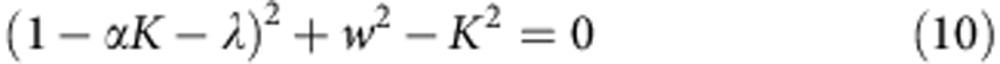


and


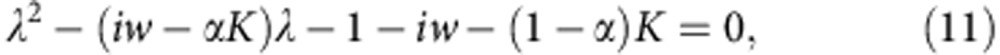


respectively. For conjugate coupling (Equation 8), AD occurs in the interval 1/*α*<*K*<*γ*(*α*), where *γ*(*α*)=(1+*w*^2^)/2 if *α*=1 and 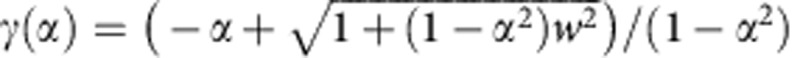 if *α*<1. Stable HSS (AD) is achieved only for *w*>1, but monotonically decreases with α, which is wiped off for *α*<*α*_min_=1/*w*. Figure 3a depicts the spread of the AD interval in the (*K*, *α*) space for *w*=5, which strictly decreases with decreasing *α* and vanishes at *α*_min_=0.2. The critical values of *α*_min_ below which the coupled oscillators retain their oscillations for all *K*>0 are shown in Fig. 3b as a function of *w*. The theoretical prediction (red line) is in good agreement with the numerical results (black squares) (Fig. 3b). For dynamic coupling Equation 9, the stable interval of HSS (AD) is 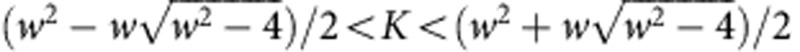 when *α*=1, which requires *w*>2 for the coupled system to experience AD. On decreasing *α*, the stable interval of HSS monotonically reduces (Fig. 3c), which ceases to exist if 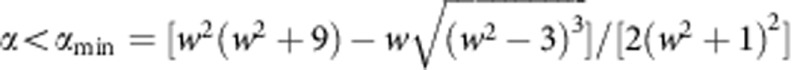. This theoretical relation (red line) agrees very well with the simulation results (black squares) as depicted in Fig. 3d. Thus, the effect of the feedback factor *α* in revoking AD to restore the natural harmonics of the coupled systems can be generally applied to distinct coupling configurations for which a stable AD is possible.

**Figure 4:**
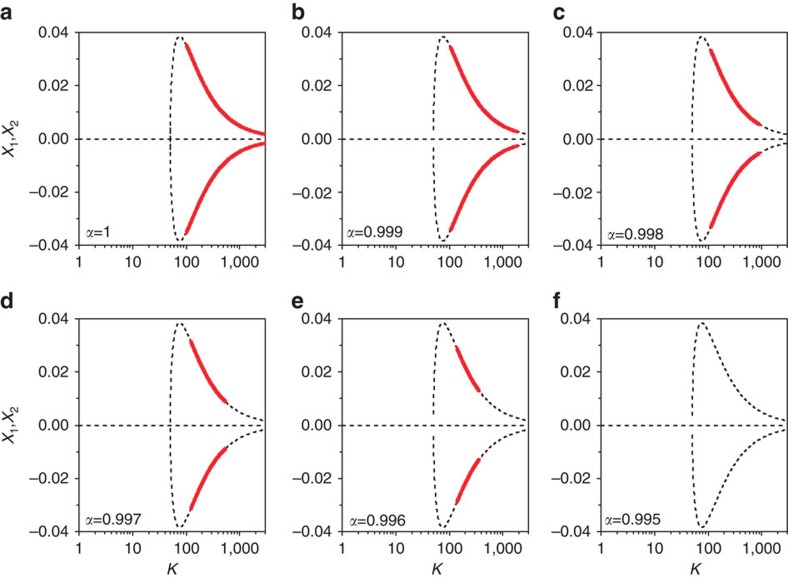
OD with one-dimensional diffusive coupling via the *x* component. (**a**-**f**) The bifurcation diagrams of the steady-state solutions for *α*=1, 0.999, 0.998, 0.997, 0.996 and 0.995, respectively. The bold red lines represent stable IHSS (OD), and the thin black lines denote unstable steady states. Even an infinitesimal change of *α* from unity drastically shrinks the stable OD interval, which shows the high efficiency of the method in restoring rhythmic activity. The frequency is used as *w*=10.

Next, we explore the capability of the feedback factor *α* in effectively retrieving rhythmic oscillations in systems where OD has been observed. Consider the following system of two Stuart–Landau oscillators via the *x* component^12^,


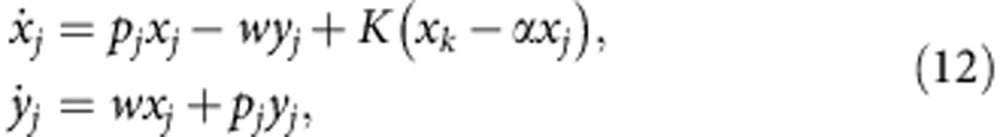


where *p*_*j*_=1−|*Z*_*j*_|^2^=1−*x*_*j*_^2^−*y*_*j*_^2^, *j*, *k*=1,2 (*j*≠*k*). Here the one-dimensional diffusive coupling involves only the real parts. This coupling form breaks the rotational symmetry of the coupled system, which is a necessary condition for the emergence of OD. Besides the HSS at the origin *Z*_1_=*Z*_2_=0 in the system (Equation 12), the system also displays an IHSS (x*, y*, −x*, −y*) that appears at *K*=(1+*w*^2^)/(1+*α*) via a pitchfork bifurcation, with 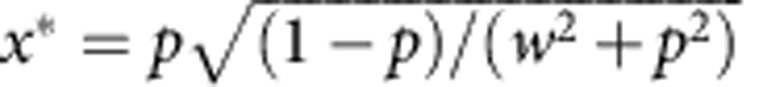 and 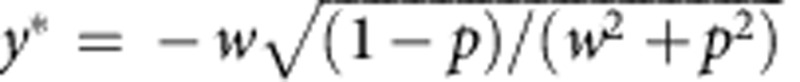 with 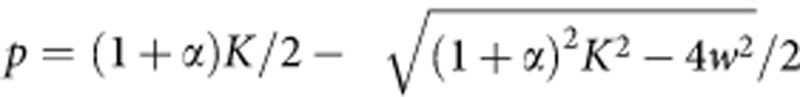. For the normal diffusive coupling, *α*=1, it was reported that the origin is unstable for all *K*>0, but the IHSS (OD) is stabilized for *K*>*w*^2^+1/4 (refs 12, 31); this is demonstrated by the bifurcation diagram plotted in Fig. 4a with *w*=10. The bold red lines denote the stable branches of the IHSS and the thin lines refer to the unstable ones. We further depict the bifurcation diagrams of the steady-state solutions for *α*=0.999, 0.998, 0.997, 0.996 and 0.995 in Fig. 4b–e, respectively. Astonishingly, we observe that even a very tiny detuning in *α* from 1 shrinks the coupling intervals of the stable IHSS (OD) (Fig. 4b–e). Moreover, no stable IHSS can be found for any coupling strength when *α*<*α*_min_≈0.995 (Fig. 4f), which attributes that OD is successfully revoked and thus oscillations are provoked in the OD regimes. The effect of the limiting factor *α* in restoration of rhythmic activity in this case is even more pronounced, since infinitesimal changes of *α* from unity lead to a sharp shrinkage of the stable IHSS regime, demonstrating the high efficiency of the proposed method in restoring oscillatory activity.

By studying diverse systems of two coupled Stuart–Landau oscillators, it has been demonstrated that incorporating the limiting feedback factor *α* in the normal diffusive coupling is a rather simple and high-efficient approach in revoking not only AD but also OD under various distinct death scenarios. Even a very minute deviation of *α* from unity drastically shrinks both AD and OD regions in the parameter space. The new coupler with *α* strengthens the tolerance to different dynamic deteriorations of coupled non-linear oscillators.

### Networked coupled Stuart–Landau oscillators

**Figure 5:**
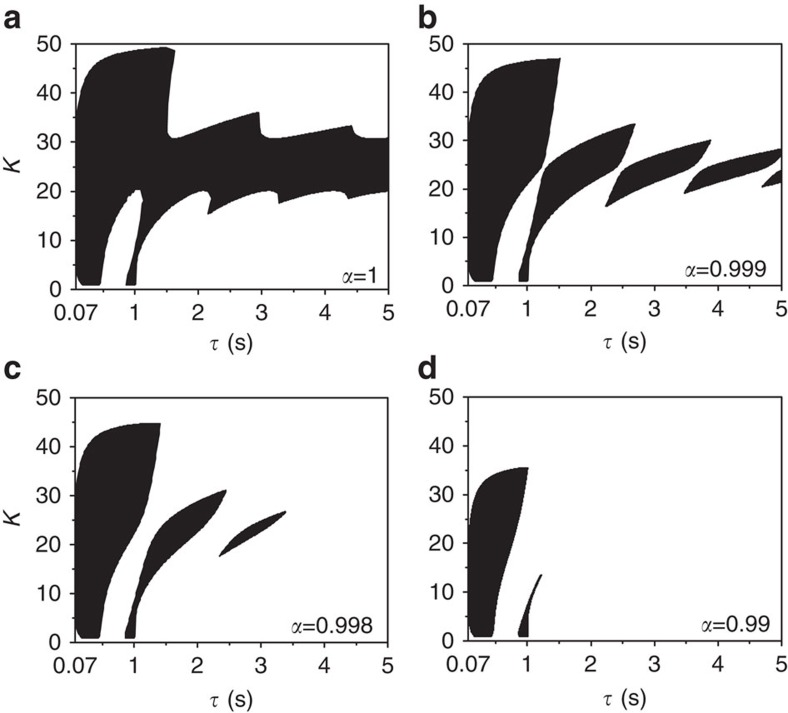
AD in an arbitrary network with distributed time delays. (**a**–**d**) The upper limiting stability region of AD as *ρ*_*N*_→0 with time delays uniformly distributed over *τ*±0.07 for different values of *α*. AD regime becomes disconnected even for a tiny decrease of *α* from 1 to 0.999. Only three AD islands survive for *α*=0.998. Decreasing *α* further, the number of AD islands decreases. The AD region completely disappears in the parameter space if *α*<*α*_min_=0.189. The intrinsic frequency is fixed as *w*=10.

The role of the diffusive feedback factor *α* in revoking AD persists for an arbitrary network of coupled Stuart–Landau oscillators. To illustrate it, let us consider a connected network of *N* Stuart–Landau oscillators with an arbitrary topology


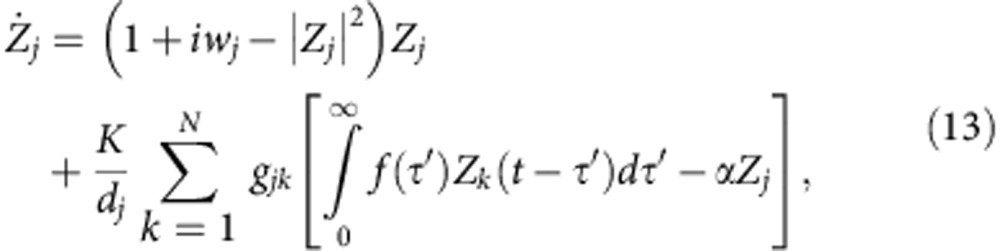


where *j*=1, 2,..., *N* and *f* is the uniformly distributed delayed kernel as in equation 7. The parameter *g*_*jk*_ determines the topology of the network, that is, *g*_*jk*_=*g*_*kj*_=1 if *j*th and *k*th nodes are linked otherwise *g*_*jk*_=*g*_*kj*_=0, *g*_*jj*_=0; and 
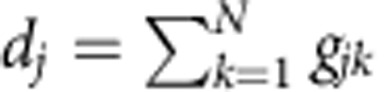
 is the *j*th node degree. The coupled system (Equation 13) (*α*=1) was studied by Atay for all-to-all networks^25^, that is, *g*_*jk*_=1 for all *j*≠*k*. Performing a linear stability analysis around the origin, the characteristic equations determining the stability of the HSS in coupled system (Equation 13) with *w*_*j*_=*w* are


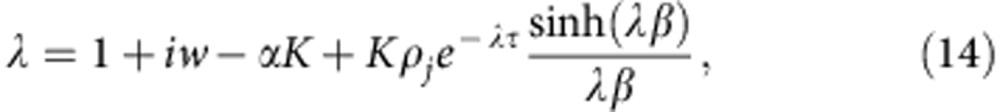


where *ρ*_*j*_'s are the eigenvalues of 
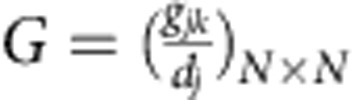
 and can be ordered as 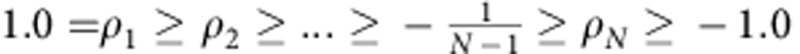 (ref. 41). The network (Equation 13) experiences AD when the largest real part of roots of equation (14) is negative for all *ρ*_*j*_. In fact, only two bounding eigenvalues *ρ*_1_=1 and −1/(*N*−1)≥*ρ*_*N*_≥−1 contribute to the stability regions of AD. The degree of the spread of the stable HSS is determined by *ρ*_*N*_; the spread of AD is larger for larger values of *ρ*_*N*_. The stable HSS regime (AD) experienced by the coupled system (Equation 13) for *α*=1 as *ρ*_*N*_→0 is plotted in Fig. 5a for uniformly distributed delays with *β*=0.07 and *w*=10. The AD regime is connected and unbounded along *τ*. We are indeed surprised to observe that the AD regime becomes disconnected even for a negligible decrease in *α*; see Fig. 5b for *α*=0.999. Decreasing *α* further, the number of AD islands decreases as shown in Fig. 5c,d for *α*=0.998 and 0.99, respectively. The AD region completely disappears in the parameter space for *α*<*α*_min_≈0.189, thereby revoking the stability of HSS (AD) in recovering oscillations in an arbitrary network with distributed time delays.

### Experiments with chemical oscillations

**Figure 6:**
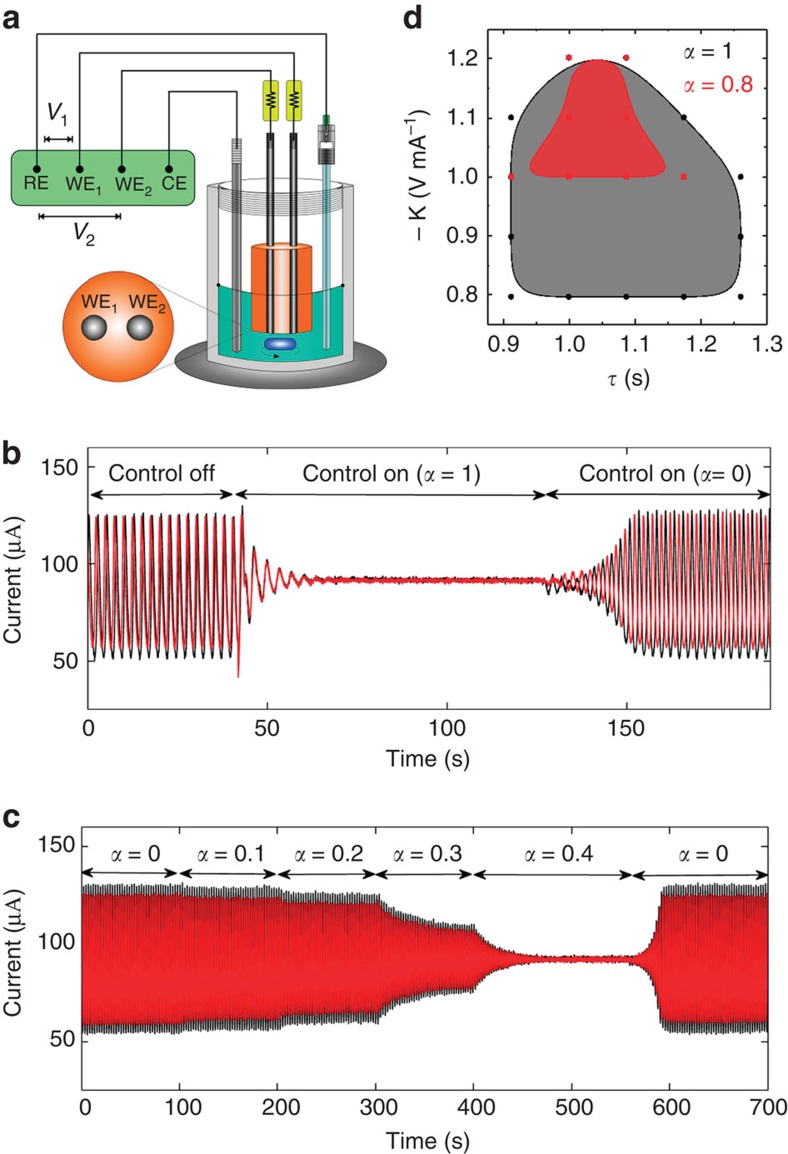
Experiments with coupled electrochemical reactions. (**a**) Schematic of the experimental setup containing the reference (RE), counter (CE) and two Ni working electrodes (WE) in an electrochemical cell with 50 ml 3 mol L^−1^ sulfuric acid at a temperature of 10 ^o^C. (**b**) Establishing AD with *α*=1 and regaining oscillations with *α*=0. Without control, the oscillations phase drift (*t*<40.13 s, *K*=0). With direct feedback control AD is observed (40.13 s<*t*<127.1 s, *K*=−1.0 V mA^−1^, *τ*=1.09 s and *α*=1). With *α*=0, the oscillations are anti-phase synchronized. (*t*>127.1 s, *K*=−1.0 V mA^−1^, *τ*=1.09 s and *α*=0). (**c**) Current time series with several values of *α*=0, 0.1, 0.2, 0.3 and 0.4. (**d**) AD domains for *α*=1 and 0.8 in the parameter space for *K* versus *τ*. The set of discrete data points are approximated by a cubic spline interpolation, which indicates the border between AD and oscillatory states.

The efficiency of the new coupling approach in revoking deaths to restore oscillations has been experimentally verified in an oscillatory chemical reaction system with electrochemical dissolution of nickel in sulfuric acidic media. Figure 6a depicts a schematic view of the experimental setup. The reactions take place on the surface of two nickel wires with diameter of 0.69 mm, spaced by 2.0 mm and embedded in epoxy resin. When an individual resistance of 2.50 kΩ are attached to each wire, and the potential *V*_0_ of each wire are set with a bipotentiostat (Bank Instruments) to 1.150 V versus Hg/Hg_2_SO_4_/sat. K_2_SO_4_ reference electrode, oscillatory current can be recorded with the built-in ammeters of the bipotentiostat between the wires and the counter electrode (1.6 mm diameter Pt coated Ti wire). This potential value is just above (by 25 mV) a Hopf bifurcation. The current oscillations of the two electrodes (*I*_1_(*t*) and *I*_2_(*t*)), shown in Fig. 6b, occur due to the non-linear kinetics of nickel dissolution in the presence of nickel-oxide films that provide hidden negative differential resistance characteristics to the electrochemical process^42^,^43^.

The interactions between the chemical reactions on the two metal surfaces are introduced by polarizing the two electrodes (indexed as *j*=1 and 2) according to the following equations:


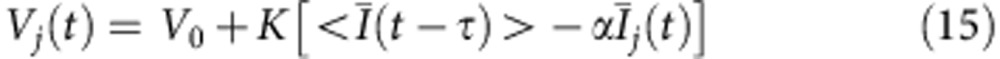


where 
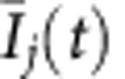
 are the offset corrected currents of the electrodes 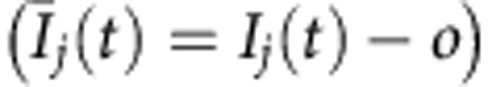, *o* is the average current overtime determined before the experiments, and 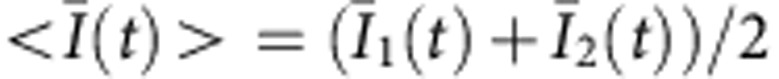 is the mean current of oscillations at time *t*. The potential perturbations, with values of *α* and *K* previously chosen with a Labview Real Time software, are applied at a rate of 1,000 Hz, which is much faster than the typical timescale of the oscillations (2.5 s).

Without control, the chemical reactions on the two electrodes take place in an oscillatory manner with slightly different periods (2.504s and 2.524 s), as shown in Fig. 6b. Because of the lack of coupling, the oscillations exhibit phase drift behaviour (Supplementary Fig. 1)^43^. When the control is turned on with *α*=1, the amplitude quickly decreases, and the system establishes a stable HSS: the delayed interaction thus causes the phenomenon of AD. The oscillations are reborn in an anti-phase configuration when *α* is adjusted to 0 for *t*>127.1 s. Corroborating with this scenario, the gradual increase of *α*, shown in Fig. 6c, clearly indicates a promoting effect on AD. For *α*<0.4, the oscillatory behaviour can be reinstated. Figure 6d depicts the AD domains for two values of *α*=1 and 0.8 in *K* versus *τ* diagram. As predicted by our theory, a small decrease of *α* markedly suppresses the AD regions.

The experimental realization of our method with chemical oscillations was successful, despite the fact that while the Stuart–Landau system is a simple, weakly non-linear, two variable model, the chemical system represents an ensemble of complex physical and chemical processes with many variables and uncertainties. The oscillations in the chemical reactions generally originate from a Hopf bifurcation, which can be well represented by the Stuart–Landau equations. Therefore, it is expected that the phenomenon of retrieving oscillations with our scheme will work for a large number of other chemical and biological systems, whose oscillatory behaviour arises as a result of a Hopf bifurcation^44^.

## Discussion

We have proposed a rather simple and efficient coupling scheme to revoke both AD and OD to retrieve rhythmic behaviours in diffusively coupled dynamical networks of non-linear oscillators. By introducing a limiting feedback factor *α* in the diffusive coupling, we have revealed that this approach is generically robust in revoking AD in coupled Stuart–Landau oscillators under distinct scenarios such as frequency mismatch, delayed coupling with both discrete and distributed time delays, and conjugate and dynamic couplings. We have shown that the OD phenomenon can also be revoked in coupled Stuart–Landau oscillators. Intriguingly, a minimal decrease of α from unity drastically reduces the size of the stable regions of both AD and OD in the parameter space. The effect of a minute deviation of *α* from unity manifests in switching the stability of the stable HSS and IHSS. Furthermore, we have experimentally confirmed the efficient role of the diffusive control factor *α* in restoring oscillations by destabilizing AD in a delay-coupled chemical reaction system with Ni electrodissolution.

The surprising effect of a minute deviation of the parameter *α* from unity could be interpreted in the following intuitive way. In the death scenarios, the normal diffusive coupling (*α*=1) brings a strong additional dissipation into the coupled oscillatory systems to suppress the oscillations. In our proposed diffusive coupling, the value of *α* describes the relation between the incoming and the outgoing flows. Specifically, the level of incoming flow is measured by *K*, whereas the outgoing one by *Kα*. Clearly, the rate of the incoming flow is greater than that of the outgoing one if 0<*α*<1, which leads to asymmetric flows in the whole-coupled systems once *α* deviates from unity. The surplus incoming flow supplies an internal energy source to compensate the dissipation of coupled systems, which serves as a driving force to induce oscillatory activity.

It is to be noted that we have corroborated our results by employing the paradigmatic Stuart–Landau oscillator, which represents a normal form describing dynamics near a supercritical Hopf bifurcation. Our findings are expected to hold true for a broad class of coupled non-linear systems near a Hopf bifurcation. In fact, we have confirmed that dynamic activity can be recovered effectively in many other diffusively coupled non-linear oscillators experiencing AD or OD, such as Brusselators (Supplementary Note 1), chaotic Lorenz oscillators (Supplementary Note 2), Pikovsky-Rabinovich circuit models (Supplementary Note 3), synthetic genetic relaxation oscillators and membrane models (Supplementary Note 4). The generality of our method has also been successfully validated in diffusively coupled dynamical networks experiencing distinctly different deteriorations of dynamic activity such as partial deaths^45^ (Supplementary Note 5) and aging transition^46^,^47^ (Supplementary Note 6). Thus, the new ingredient *α* in the coupling serves as a very general framework to strengthen the robustness of dynamic activity in diffusively coupled networks.

Our proposed coupling scheme has important consequences in particular in biology. Recent studies of synthetic biology have made significant progress in the construction of populations of intracellular oscillators that mimic naturally occurring clocks as a means to gain insight about the functionality of biological networks^48^,^49^. In this regard, it would be beneficial to develop sensing compartments, which are non-difference (*α*=0) for maintaining the oscillations. Assuming that the intrinsic dynamics of a cell is oscillatory, this means that it is important to develop isolated reference states inside the cells where the periodic variations of the cell life cycle are less affected. In neural networks, two major types of coupling forms are: gap junctions and chemical synapses^50^. Gap junctions are based on the potential difference between the cells (*α*=1), while chemical synapses are through substances generated by action potential and thus do not directly depend on the potential difference (*α*=0). Our prediction indicates that chemical synapses play a crucial role in maintaining the oscillations, and by changing the ratio of gap junction and electrical coupling could be a versatile way to tune the extent of oscillations in the networks. Our study also has important implications for the design of coupling schemes for synchronization engineering, that is, for tuning the phase relationship between oscillatory units. Feedback-based algorithms can be applied directly or through difference schemes^51^. In general, to avoid AD, direct feedback methods could provide wider parameter ranges at strong feedback gains where synchronization structures are to be attained without amplitude suppression^52^.

A population of coupled non-linear oscillators are deemed to serve as an efficient tool to enhance our understanding of collective dynamics spontaneously emerging in real-life systems. The coupling in an ensemble of non-linear oscillators could be designed in different ways. In particular, diffusive coupling is a natural and fundamental type of interaction, which has attracted great interests in different context in a wide range of disciplines including physics, chemistry and biology for many decades. Communication between cells and most of biological process occur through diffusion of ions. In chemical reactions, mixing of different reactants generally involves diffusive interactions. Therefore, in this work, we have considered the diffusive coupling as a starting point of our study in revealing restoration of oscillations of coupled networks. By introducing a new parameter modifying the nature of the normal diffusive coupling, we have elaborated that even a very tiny deviation from the traditional diffusive coupling causes a drastic influence on the dynamic activity of coupled dynamical systems. Our results illuminate the role of diffusive coupling in the generation of sustained rhythmic activity in real-world systems. It was reported that the phenomena of AD and OD are also possible for coupled non-linear systems with non-diffusive couplings^53^,^54^,^55^,^56^,^57^. How to revoke AD and OD in non-diffusively coupled systems constitutes our next task to be studied.

Finally, our proposed coupling scheme can be easily realized not only in different model studies but also for diverse experimental setups. We firmly speculate that our approach is feasible in other distinct experiments, where AD or OD has been reported^58^,^59^,^60^,^61^. The non-trivial influence of the new diffusive coupling scheme on the qualitative properties of dynamical systems indicates a new path for future studies of other collective behaviours in diffusively coupled non-linear oscillators such as chimera states^62^,^63^, explosive synchronization^64^,^65^ and glass states^66^,^67^,^68^. The framework of our study sheds significantly new insights on the diffusive coupling in manipulating oscillatory dynamics of coupled complex non-linear systems, which will have a strong impact and invoke wide interests in the field of complex systems science as well as in various applications from biology via engineering to social sciences.

## Methods

### Numerical simulations

All numerical integrations of coupled dynamical equations are performed by employing a standard explicit fourth-order Runge–Kutta method with integration steps *h*=0.01 and 0.001, respectively. There are no qualitative differences between two different steps. Random initial conditions can be adopted for AD in coupled Stuart–Landau oscillators, which means that AD is not only linearly but also globally stable in the phase space. In contrast, as OD always coexists with stable limit-cycle motions, initial conditions near the IHSS are used for OD, or many different random initial conditions are tried until reaching OD.

### Steady-state solutions for AD and OD

The onset of AD in coupled Stuart–Landau oscillators implies that the unstable origin is stabilized by the coupling. Thus the steady-state solution for AD in coupled Stuart–Landau oscillators is HSS at *Z*_*j*_=0. In contrast, OD manifests due to the appearance of stable IHSS. In general, for OD in coupled non-linear oscillators, the Newton–Raphson algorithm^69^ is used to locate the IHSS solution. In coupled Stuart–Landau oscillators in equation (12), the IHSS solution for OD is analytically derived, which is also confirmed by the Newton–Raphson algorithm.

### Linear stability analysis

The occurrences of AD and OD are verified from a standard linear stability analysis after obtaining steady-state solutions of AD and OD in coupled Stuart–Landau oscillators. AD (OD) is stable, if and only if all the real parts of resulted characteristic eigenvalue equations are negative. Once a time delay is involved, the corresponding characteristic eigenvalue equations are transcendental, which have infinitely many (both complex and real) roots with negative real parts, but only a finite number of roots with positive real parts attributed to instability. Generally, the characteristic roots of transcendental characteristic equations cannot be solved analytically. We numerically compute the largest real part of characteristic roots with the pseudospectral differentiation techniques^70^. For one special case, the characteristic roots can be expressed explicitly using the Lambert function *W* as indicated in equation (4).

### AD boundaries

The AD boundaries in equation (5) are analytically derived. The analysis is based on the fact that as tuning *τ* or *K*, the stability of HSS in coupled Stuart–Landau oscillators may be switched only if a characteristic eigenvalue root *λ* in equation (3) crosses the imaginary axis. For the critical situation, we have *λ*=*i*Ω. Inserting into equation (3) and separating into real and imaginary part, the AD boundary curves can be obtained after some straightforward algebraic manipulations.

### The critical value of *α*_min_

The values of *α*_min_ theoretically predicted in equation (6) are from the intersection condition of AD boundaries in equation (5). Generally, the threshold value of *α*_min_ in all cases is calculated by meticulously decreasing *α* from 1 until the stable regime of HSS (AD) or IHSS (OD) is extinct in the whole parameter space.

## Additional information

**How to cite this article**: Zou, W. *et al*. Restoration of rhythmicity in diffusively coupled dynamical networks. *Nat. Commun.* 6:7709 doi: 10.1038/ncomms8709 (2015).

## Supplementary Material

Supplementary Information Supplementary Figures 1-8, Supplementary Notes 1-6 and Supplementary References
