# Supplementary material for: Restoration of rhythmicity in diffusively coupled dynamical networks
Source: Nat Commun. 2015 Jul 15;6:7709. doi: 10.1038/ncomms8709 (PMC4518287; doi:10.1038/ncomms8709)
Supplement: Supplementary Information — Supplementary Figures 1-8, Supplementary Notes 1-6 and Supplementary References [file ncomms8709-s1.pdf]

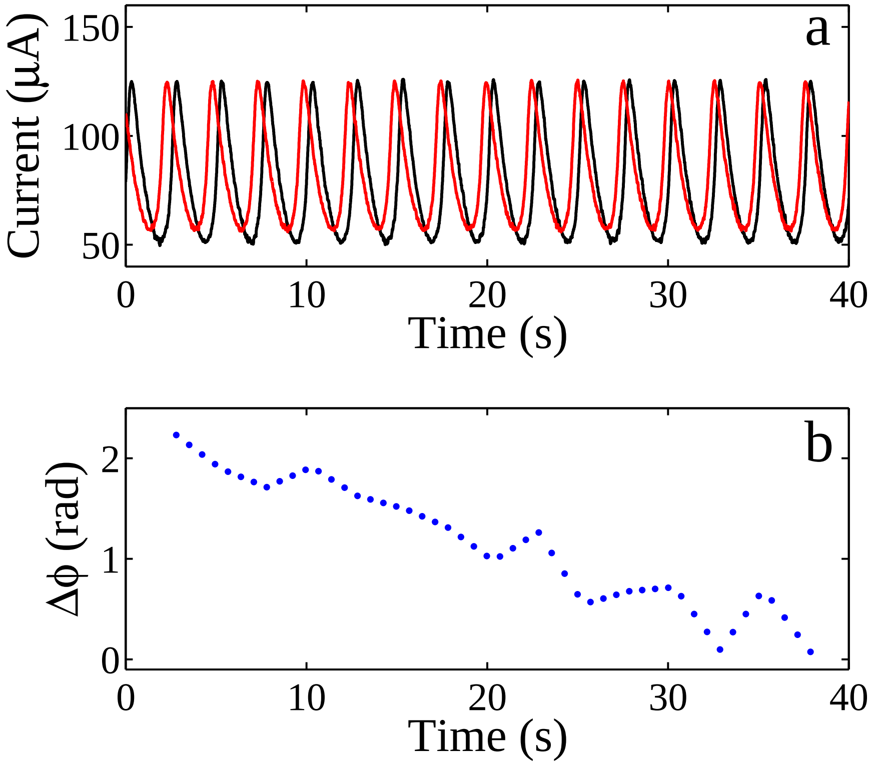

Supplementary Figure 1: **Phase drift of the chemical oscillations without coupling.** **(a)** Current oscillations at $V_0 = 1.15V$ in the anodic dissolution of nickel for electrode 1 (black) and electrode 2 (red). Time-series corresponding to the uncoupled portion of Fig. 6(b) of the main text. **(b)** The phase difference $\Delta\phi = \phi_1 - \phi_2$, obtained with the linear interpolation method [1], as a function of time.

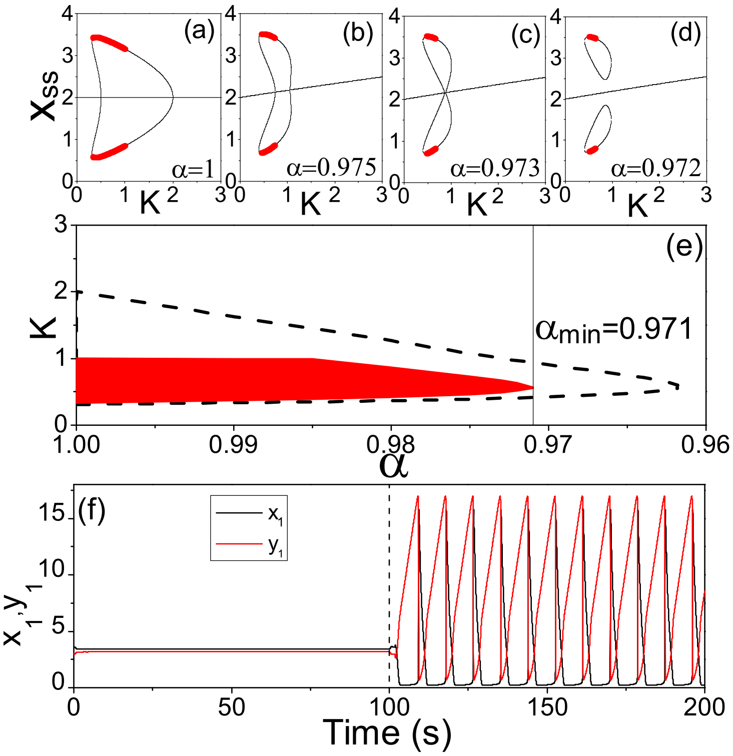

Supplementary Figure 2: **Two coupled Brusselators. (a)-(d)** The bifurcation diagrams of the steady-state solutions for $\alpha = 1$, 0.975, 0.973, and 0.972, respectively. The bold red lines represent stable steady states, and the thin black lines denote unstable ones. **(e)** The interval of strength for stable OD (red regions) vs α. The black dashed line corresponds to the existence condition for IHSS. **(f)** The plot of time series of one Brusselator. Limit-cycle oscillations are regained as α is switched from 1 to 0.97 at $t = 100$. The coupling strength is fixed at $K = 0.5$.

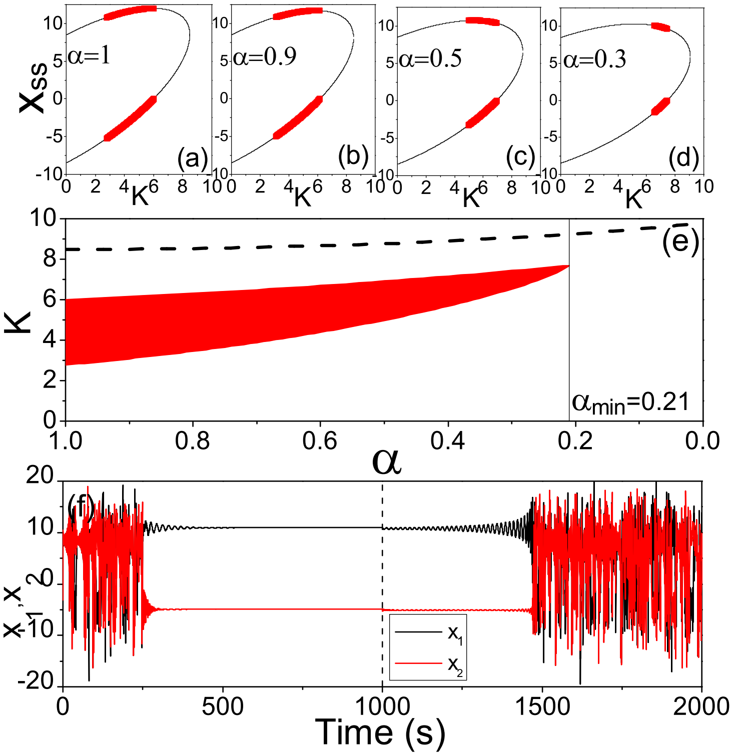

Supplementary Figure 3: **Two coupled chaotic Lorenz oscillators. (a)-(d)** The bifurcation diagrams of the IHSS of x-component for $\alpha = 1$, 0.9, 0.5, and 0.3, respectively. The bold red lines represent stable IHSS of x_1^* and x_2^*, and the thin black lines denote unstable ones. **(e)** The stable coupling interval of OD (red regions) vs α. The black dashed line denotes the largest coupling strength $K_{\max}$ for the emergence of IHSS $A_{1,2}$. **(f)** The plot of time series of x-component for two coupled Lorenz oscillators. Chaotic oscillations are restored from OD as α is switched from 1 to 0.9 at $t = 1000$. The coupling strength is fixed at $K = 3$.

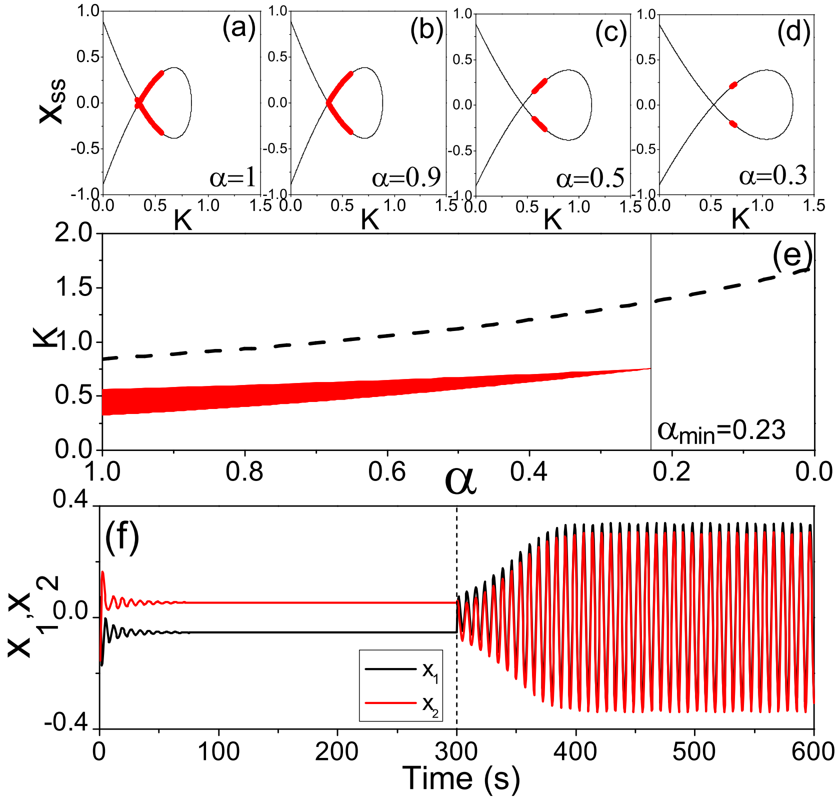

Supplementary Figure 4: **Two coupled Pikovsky-Rabinovich circuit models.** **(a), (b), (c), and (d)** The bifurcation diagrams of the IHSS of x-component for $\alpha = 1$, 0.9, 0.5, 0.3, respectively. The bold red lines represent stable IHSS of x_1^* and x_2^*, and the thin black lines denote unstable ones. **(e)** The stable coupling interval of OD (red regions) vs α. The black dashed line represents the largest coupling strength K_{max} for the emergence of IHSS $A_{1,2}$. **(f)** The plot of time series of x-component for two coupled Pikovsky-Rabinovich circuit models, where the value of α is switched from 1 to 0.8 at $t = 300$. Self-oscillations die out for the normal diffusive coupling with $\alpha = 1$, which are clearly recovered with $\alpha = 0.8$. The coupling strength is fixed at $K = 0.37$.

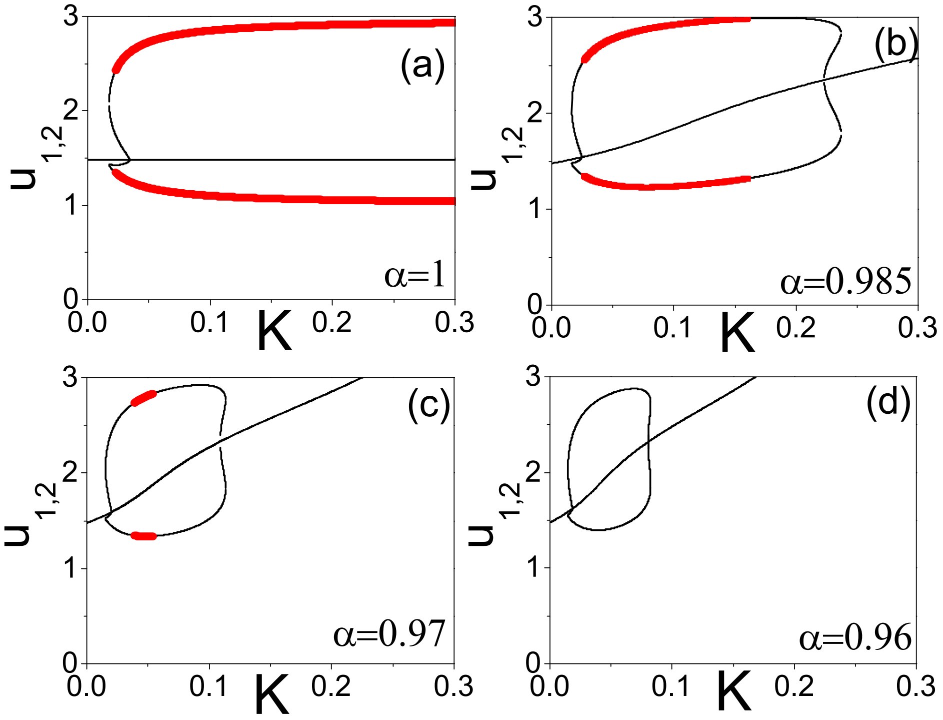

Supplementary Figure 5: **Coupled synthetic genetic relaxation oscillators. (a), (b), (c), and (d)** The bifurcation diagrams of the steady-state solutions for different values of α, where the red bold lines represent stable IHSS and the black thin lines denote unstable ones.

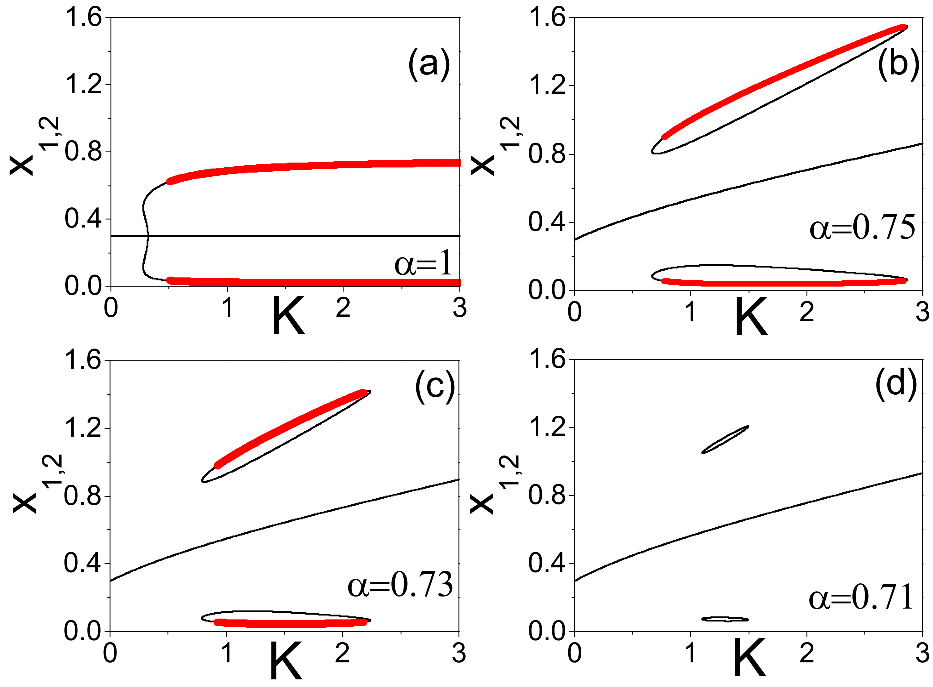

Supplementary Figure 6: **Coupled cell membrane models. (a), (b), (c), and (d)** The bifurcation diagrams of the steady-state solutions for different values of α, where the red bold lines represent stable IHSS and the black thin lines denote unstable ones.

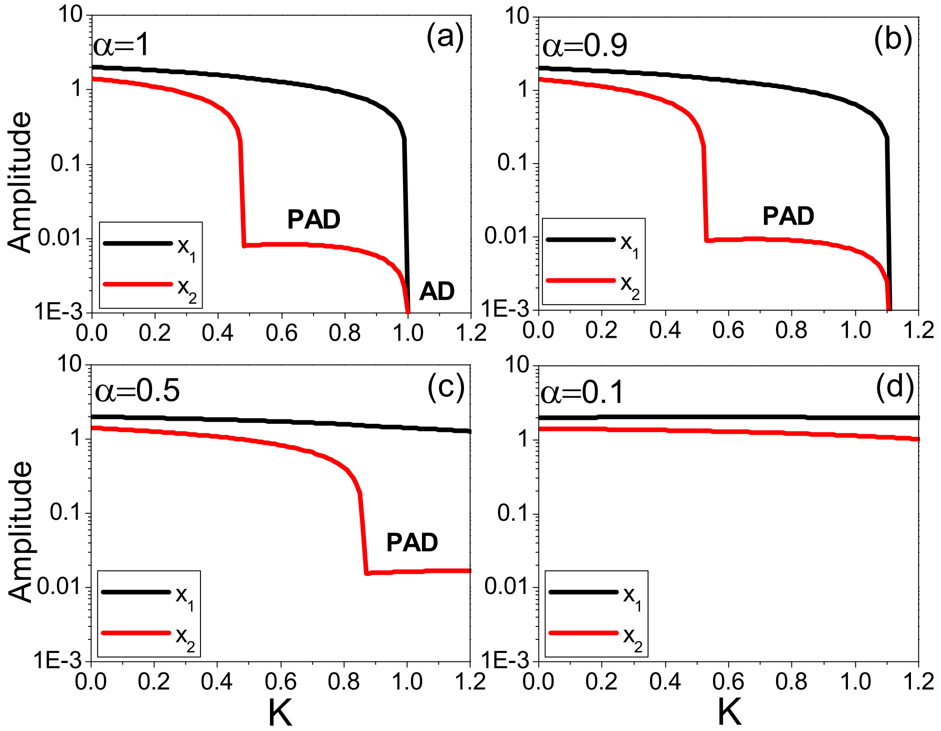

Supplementary Figure 7: **Coupled Van der Pol oscillators. (a), (b), (c), and (d)** The amplitudes of x_1 and x_2 against the coupling strength K for $\alpha = 1$, 0.9, 0.5, and 0.1, respectively. The region marked by PAD corresponds to partial AD, in which only the second oscillator is quenched with a negligible fluctuation while the first one continues to exhibit high-amplitude oscillations. As decreasing α from 1, both AD and PAD are revoked and high-amplitude oscillations for both oscillators are recovered. The vertical scale is logarithmic.

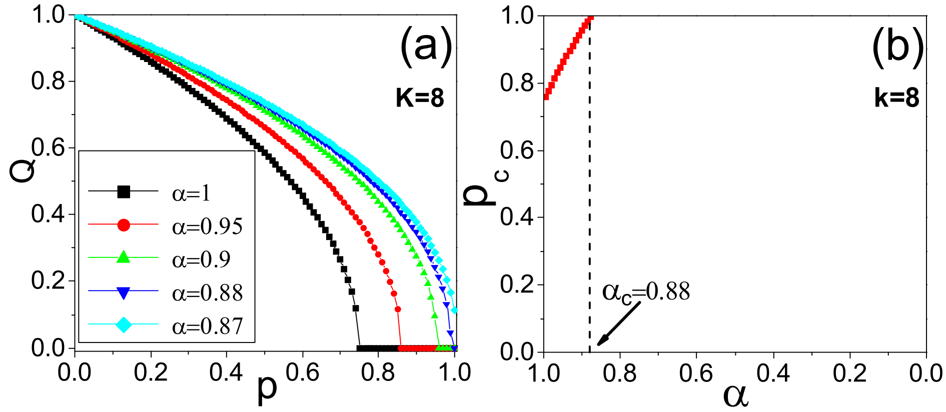

Supplementary Figure 8: **Coupled Stuart-Landau oscillators with aging transition.** **(a)** The normalized order parameter $Q = |\bar{Z}(p)|/|\bar{Z}(p=0)|$ against the ratio p of inactive oscillators for various values of α. With increasing of p from zero, the global oscillation of coupled oscillator network vanishes at p_c ($Q = 0$). The critical value of p_c increases and reaches at $p_c = 1$ with decreasing of α from unity to a certain value of α_c. For $\alpha < \alpha_\mathrm{c}$, Q is nonzero even for $p = 1$. **(b)** The critical value of p_c monotonically increasing with decreasing α from unity. The dynamic activity of coupled network persists even for all elements turning inactive ($p = 1$) if $\alpha < \alpha_\mathrm{c}$. The value of $K = 8$ is used.

Supplementary Note 1: Coupled Brusselators

An initial observation of OD (a stable IHSS) was in a system of two coupled Brusselators by Prigogine and Lefever [2]. Further, Bar-Eli performed a detailed study of OD in coupled Brusselators [3]. We will demonstrate that the feedback factor α can effectively revoke the stability of OD (a stable IHSS) to retrieve limit-cycle oscillations in the following two coupled Brusselators [3],

$$\begin{aligned} \dot{x}_j &= -(B+1)x_j + x_j^2 y_j + A + K(x_k - \alpha x_j), \\ \dot{y}_j &= Bx_j - x_j^2 y_j + K(y_k - \alpha y_j), \end{aligned} \tag{1}$$

where $j, k = 1, 2$ $(j \neq k)$. For $K = 0$, the single uncoupled Brusselator has a steady state at ($x^* = A$, $y^* = B/A$), which loses stability via a supercritical Hopf bifurcation as $B > A^2 + 1$ and stable limit-cycle appears; here we fix $B = 10$ and $A = 2$. OD in the coupled system (1) for $\alpha = 1$ has been studied in great detail by Bar-Eli [3]. For $\alpha = 1$, Bar-Eli [3] has shown that the HSS is always unstable for any K. However, a new IHSS can be created and stabilized in a pronounced interval of K (bold red lines) as illustrated in Supplementary Figure 2(a) for $\alpha = 1$. With a tiny decrease of α from 1, interestingly, the structure and the stability of both IHSS and HSS are tailored. The bifurcation diagrams of the steady states of the coupled system (1) for $\alpha = 0.975$, 0.973, and 0.972 are depicted in Supplementary Figures 2(b)-2(d), respectively. Clearly, the solution structure of both HSS and IHSS deforms and the interval of stable IHSS monotonically decreases with α. It should be emphasized that OD emerges due to the stabilization of IHSS, which appears via a pitchfork bifurcations of the unstable steady state ($x^* = A$, $y^* = B/A$). Supplementary Figure 2(e) depicts the range of K with stable IHSS as a function of α, where the dashed line indicates the limit of K for the emergence of stable IHSS (OD). It is evident that no stable IHSS can be found for $\alpha < \alpha_{\text{min}} = 0.971$. Supplementary Figure 2(f) illustrates the time series of one of two coupled Brusselators (1) for $K = 0.5$. The value of α is switched from 1 to 0.97 at $t = 100$, which signals that OD is indeed revoked and limit-cycle oscillations are provoked. The presence of control parameter α may be a crucial ingredient for the emergence of sustained temporal oscillations in autocatalytic reactions of coupled chemical systems.

Supplementary Note 2: Coupled chaotic Lorenz oscillators

The coupling approach with the limiting factor α also provides a simple means to revoke OD and regain oscillations in coupled chaotic nonlinear oscillators. We analyze the following two coupled chaotic Lorenz oscillators [4]

$$
\begin{aligned}
\dot{x}_j &= \sigma(y_j - x_j), \\
\dot{y}_j &= r x_j - y_j - x_j z_j, \\
\dot{z}_j &= x_j y_j - b z_j + K(x_k - \alpha x_j),
\end{aligned} \tag{2}
$$

where $j, k = 1, 2$ $(j \neq k)$. With a typical parameter set of ($\sigma = 10$, $r = 28$, $b = 8/3$), each uncoupled Lorenz oscillator ($K = 0$) has a well-known chaotic attractor and two symmetric unstable equilibria $A_{\pm}(\pm\sqrt{b(r-1)}), \pm\sqrt{b(r-1)}), r-1)$ [4]. For two coupled lorenz oscillators (2), a set of new IHSS $A_{1,2}(x_1^*, x_1^*, r-1, x_2^*, x_2^*, r-1)$ stemmed from $A_{\pm}$ can be induced by the coupling strength K, where $x_1^* = K(1+\alpha)/2 + \sqrt{b(r-1) - [1-(\alpha-1)^2/4]K^2}$ and $x_2^* = K(1+\alpha) - x_1^*$. Clearly, the IHSS $A_{1,2}$ approaches to (A_+, A_-) as $K \to 0$, which exists for $K < K_{\text{max}}(\alpha) = \sqrt{b(r-1)/[1-(\alpha-1)^2/4]}$. For $\alpha = 1$, the IHSS $A_{1,2}$ is stabilized in an appropriate interval of K as indicated by bold red lines in Supplementary Figure 3(a). We further plot the bifurcation diagrams of the IHSS of x-component for two coupled Lorenz oscillators (2) with $\alpha = 0.9$, 0.5, and 0.3 in Supplementary Figures 3(b)-3(d), respectively. Interestingly, it is found that the solution structure of IHSS is nearly undeformed upon decreasing the value of α. But the length of coupling interval K for stable IHSS (bold red lines) strictly decreases. Note that, unlike two coupled Brusselators, here OD is realized by stabilizing new IHSS emanating from two unstable steady states $A_{\pm}$. Supplementary Figure 3(e) shows the dependence of the stable OD interval on α, where the dashed line limits the largest value K_{max} of coupling strength K for the existence of IHSS $A_{1,2}(\alpha)$. The IHSS $A_{1,2}(\alpha)$ is unstable for any possible $K < K_{\text{max}}(\alpha)$ if $\alpha < \alpha_{\text{min}} = 0.21$. We depict the time series of x-component for two coupled Lorenz oscillators (2) with $K = 3$ in Supplementary Figure 3(f). Clearly, chaotic motions die out for the normal diffusive coupling with $\alpha = 1.0$, where the two Lorenz oscillators are attracted to different fixed points in the phase space for $t < 1000$; i.e., OD occurs. However, OD is revoked and chaotic behavior is revived by slightly tuning α from 1 to 0.9. The new ingredient α in the diffusive coupling, which maintains robust chaos, opens valuable possibility for potential applications in secure communication and chaos control.

Supplementary Note 3: Coupled Pikovsky-Rabinovich circuits

The validness of our coupling approach in revoking OD is further confirmed in the following two coupled Pikovsky-Rabinovich circuit models [5]:

$$\begin{aligned}\dot{x}_j &= y_j - \beta z_j, \\ \dot{y}_j &= -x_j + 2\gamma y_j + \eta z_j + K(y_k - \alpha y_j), \\ \dot{z}_j &= (x_j - z_j^3 + z_j)/\mu, \end{aligned} \quad (3)$$

where $j \neq k$, $j, k = 1, 2$, $\beta = 1.0$, $\gamma = 0.26$, $\eta = 0.15$, and $\mu = 0.4$. For $K = 0$, each Pikovsky-Rabinovich oscillator has a stable chaotic attractor and two symmetric unstable equilibria $A_\pm$ $(z_\pm^{*3} - z_\pm^*, \beta z_\pm^*, z_\pm^*)$ with $z_\pm^* = \pm\sqrt{1 + 2\gamma\beta + \eta}$. For $K \neq 0$, a set of IHSS stemmed from $A_\pm$ is $A_{1,2}(z^{*3} - z^*, \beta z^*, z^*, -z^{*3} + z^*, -\beta z^*, -z^*)$, where $z^* = \sqrt{1 + 2\gamma\beta + \eta - (1+\alpha)\beta K}$. Obviously, the IHSS $A_{1,2}$ approaches to (A_+, A_-) as $K \to 0$, which exists for $K < K_{\text{max}} = (1 + 2\gamma\beta + \eta)/[(1 + \alpha)\beta]$. We plot bifurcation diagrams of IHSS of x-component with $\alpha = 1$, 0.9, 0.5, and 0.3 in Supplementary Figures 4(a)-4(d), respectively. Clearly, the coupling interval K for stable IHSS (bold red lines) decreases with decreasing α from 1. We further depict the dependence of the interval of stable IHSS (OD) on α in Supplementary Figure 4(e), where the dashed line limits the largest value K_{max} of coupling strength K for the existence of IHSS $A_{1,2}(\alpha)$. The IHSS $A_{1,2}(\alpha)$ is unstable for any possible $K < K_{\text{max}}(\alpha)$ if $\alpha < \alpha_{\text{min}} = 0.23$. We plot the time series for the two variables of x_1 and x_2 in Supplementary Figure 4(f) with the coupling strength $K = 0.37$, where the value of α is switched from 1 to 0.8 at $t = 300$. We observe that OD appears for the normal diffusive coupling with $\alpha = 1$, which is successfully revoked by adjusting the value of α from 1 to 0.8. We speculate that our scheme in revoking OD to restore self-sustained oscillations can be corroborated in the experimental observations of coupled nonlinear circuits.

Supplementary Note 4: Coupled biological oscillators

The proposed coupling approach is also applicable to biologically relevant systems. We will confirm the validness of our results in biological networks of coupled synthetic genetic relaxation oscillators and membrane models, where OD has been reported in both systems. The time evolution of two coupled synthetic genetic relaxation oscillators is governed by the dimensionless equations [6, 7]

$$\begin{aligned}\dot{u}_i &= \alpha_1 f(v_i) - u_i + \alpha_3 h(w_i),\\ \dot{v}_i &= \alpha_2 g(u_i) - v_i,\\ \dot{w}_i &= \epsilon(\alpha_4 g(u_i) - w_i) + K(w_j - \alpha w_i),\end{aligned} \tag{4}$$

where $i \neq j$, $i, j = 1, 2$. Here u_i and v_i represent the proteins of which the toggle switch is made in the ith cell, and w_i represents the concentration of autoinducer molecules which diffuse through the cell membrane. The dimensionless parameters α_1 and α_2 regulate the operation of the repressor in the toggle switch, α_3 is the activation due to the autoinducer, and α_4 is the repressing of the autoinducer. The presence of multiple time scales in the model (established for $\epsilon \ll 1$) allows the system to produce relaxation oscillations. The corresponding functions are: $f(v_i) = 1/(1 + v_i^{\beta})$, $g(u_i) = 1/(1 + u_i^{\gamma})$, and $h(w_i) = w_i^{\eta}/(1 + w_i^{\eta})$. The values of parameters are fixed as $\epsilon = 0.01$, $\alpha_1 = 3$, $\alpha_2 = 5$, $\alpha_3 = 1$, $\alpha_4 = 4$, and $\beta = \eta = \gamma = 2$. Supplementary Figures 5(a)-5(d) illustrate the bifurcation diagrams of steady-state solutions of the coupled system (4) for $\alpha = 1$, 0.985, 0.97, and 0.96, respectively, where the thick (red) lines represent the stable steady states and the thin (black) unstable ones. Clearly the solution structure of IHSS deforms and the interval of stable IHSS (OD) monotonically decreases with decreasing α. OD is impossible when α is below a critical value α_{min}, which asserts the effective role of the feedback factor α in revoking OD in coupled synthetic genetic relaxation oscillators.

We have also confirmed our results in the model used to describe kinetics of lipid peroxidation in a cell membrane. The reduced dimensionless form for such two coupled oscillators is described as [8]

$$\begin{aligned}\dot{x}_i &= \frac{1}{\epsilon}(k + 0.5x_i y_i - x_i^2 - \frac{1.5\gamma x_i}{x_i + \delta}),\\ \dot{y}_i &= \eta - 1.5x_i y_i - d y_i - \frac{1.5\gamma x_i}{x_i + \delta} + K(y_j - \alpha y_i),\end{aligned} \tag{5}$$

where $i \neq j$, $i, j = 1, 2$. Here, the parameter ϵ controls the stiffness of the oscillators and could vary between 0.01 and 0.1. k, γ, d, δ, and η are the rates of influx

and efflux of the participants. The values of the parameters are fixed as $\epsilon = 0.05$, $k = 0.05$, $\gamma = 0.5$, $\eta = 2.5$, $\delta = 0.15$, and $d = 0.2$. The bifurcation diagrams of the steady-state solutions with different values of α are depicted in Supplementary Figure 6. Again, we find that the solution structure of IHSS deforms and the interval of stable IHSS (OD) monotonically shrinks as decreasing α from 1, which no longer exists as $\alpha < \alpha_{\text{min}}$.

With the above demonstrations of our approach in two realistic models of coupled biological oscillators, we would like to emphasize that the diffusive coupling with the new ingredient α has a strong implication for sustaining rhythmic activity in coupled biological systems.

Supplementary Note 5: Coupled Van der Pol oscillators

When the individual units in the coupled systems are sufficiently different, there is a possibility that only part of elements are quenched while the others oscillate even though they remain connected. This phenomenon was termed as partial AD (PAD) [9]. We will demonstrate that the coupling approach with α can also recover the global oscillations of systems experiencing PAD. Consider a pair of coupled Van der Pol oscillators [9]:

$$\ddot{x}_j + \varepsilon(x_j^2 - a_j)\dot{x}_j + w_j^2 x_j = \varepsilon K(\dot{x}_k(t-1) - \alpha\dot{x}_j), \tag{6}$$

where $j \neq k$, $j, k = 1, 2$, $a_1 = 1$, $a_2 = 0.5$, $w_1 = 0.1$, $w_2 = 1$, and $\varepsilon = 0.1$. In the absence of coupling $(K = 0)$, the jth Van der Pol oscillator has an attracting limit cycle solution and an unstable steady state at the origin. It was reported that for the normal diffusive coupling with $\alpha = 1$, AD occurs for $K > 1$, for which both oscillators are completely quenched [9]. On the other hand, a PAD occurs for $0.5 < K < 1$, for which only the second oscillator is damped with a negligible fluctuation and the first one continues to exhibit pronounced oscillations [9]. Supplementary Figures 7(a)-7(d) depict the amplitudes of x_1 and x_2 vs the coupling strength for different values of $\alpha = 1$, 0.9, 0.5, and 0.1, respectively. The interval marked by PAD denotes the range of coupling strength for the onset of partial AD in the coupled systems, where only the second oscillator is suppressed independent of the first one although they are connected. With decreasing α from 1, PAD is revoked and the global oscillations with large amplitudes are recovered. Intriguingly, AD first transits to PAD, then PAD is revoked and pronounced oscillations for both oscillators are regained. Thus, the new ingredient α in the coupling efficiently produces high-amplitude oscillations for all units in the coupled systems experiencing not only AD but also PAD, which implies that the control parameter α is quite robust in recovering rhythmic behaviors of the coupled systems experiencing different dynamic deteriorations.

Supplementary Note 6: Coupled Stuart-Landau oscillators with aging transition

A system of coupled oscillator networks loses its oscillatory activity when the ratio p of the inactive elements to the active ones is progressively raised from zero to exceed a certain threshold p_c. If the coupling strength K is beyond a critical value K_c, the threshold p_c is less than unity. This phenomenon was termed as an aging transition [10]. We will illustrate that the presence of the new ingredient α in the diffusive coupling efficiently recovers the global oscillation of coupled system with an aging transition. Consider the following globally and diffusively coupled Stuart-Landau oscillators of the form,

$$\dot{Z}_j \;=\; (A_j + iw - |Z_j|^2)Z_j + \frac{K}{N}\sum_{k=1}^{N}(Z_k - \alpha Z_j), \qquad (7)$$

for $j = 1, 2, ..., N$, A_j specifies the distance from a Hopf bifurcation. Without coupling $K = 0$, the jth element exhibits limit-cycle oscillations $Z_j = \sqrt{A_j}e^{iwt}$ if $A_j > 0$, and is attracted to the trivial fixed point $Z_j = 0$ if $A_j < 0$. Daido and Nakanishi [10] defined that the aging of the coupled system proceeds in such a way that an active oscillator with $A_j > 0$ turns inactive with $A_j < 0$. Without loss of generality, one can set the group of active elements to be $j = 1, 2, ..., N(1-p)$ with $A_j = a = 2$ and that of inactive elements to be $j = N(1-p)+1, ..., N$ with $A_j = b = -1$, where p is the fraction of inactive elements in the network. We fix the system size at $N = 1000$, which is large enough to enable one to treat the ratio p as a continuous parameter. The other parameters are used as $a = 2$, $b = 1$, and $w = 3$.

For the normal diffusive coupling with $\alpha = 1$, Daido and Nakanishi [10] showed that the coupled network loses its macroscopic oscillation if $p > p_c = a(K+b)/[(a+b)K]$ and $K > a$. Such a transition can be identified from a system order parameter $|\bar{Z}| = |N^{-1}\sum_{k=1}^{N} Z_k|$, whose value measures the level of global oscillation of the coupled network. Supplementary Figure 8(a) depicts its normalized value $Q = |\bar{Z}(p)|/|\bar{Z}(p=0)|$ against p for some values of α with a fixed coupling strength at $K = 8$. With increasing of p from zero, Q decreases from 1 to 0 at p_c, i.e., the global oscillation vanishes for $p > p_c$. The larger value of p_c means the lager ratio of inactive oscillators for the coupled network transiting from the global oscillatory behavior to a quiescent state. The value of p_c serves as an index to monitor the dynamical robustness of the network [10, 11]; and the higher the value of p_c, the network will be more robust. Supplementary Figure

8(b) further plots the dependence of p_{c} on α. It can been clearly seen that, with decreasing α from 1, p_{c} monotonically increases and rapidly reaches at $p_{\mathrm{c}} = 1$ for $\alpha_{\mathrm{c}} = 0.88 \gg 0$. Surprisingly, it can be found that p_{c} greatly increases even as slightly decreasing α from 1, which implies the high efficiency of the method in recovering dynamic behavior of the coupled systems. Remarkably, the value of Q is nonzero even for $p = 1$ with further decreasing α below α_{c}, which illustrates that the global oscillation is sustained even for all elements turning inactive; the dynamic activity of coupled network is robust to any level of inactivation of units if $\alpha < \alpha_{\mathrm{c}}$. The similar phenomenon is observed for other values of coupling strength $K > a$. Thus, the coupling approach with α provides a simple and efficient means to recover global oscillation in diffusively coupled oscillator networks that has been lost due to some inactivate elements. The proposed coupling scheme with the limiting factor α plays a positive role in strengthening the robustness of dynamic activity in diffusively coupled systems.

Supplementary References

[1] Pikovsky, A., Rosenblum, M. & Kurths, J. *Synchronization: A Universal Concept in Nonlinear Sciences* (Cambridge University Press, Cambridge, 2001).

[2] Prigogine, I. & Lefever, R. Symmetry breaking instabilities in dissipative systems. *J. Chem. Phys.* **48**, 1695-1700 (1968).

[3] Bar-Eli, K. On the stability of coupled chemical oscillators. *Physica D* **14**, 242-252 (1985).

[4] Lorenz, E. N. Deterministic nonperiodic flow. *J. Atmos. Sci.* **20**, 130-141 (1963).

[5] Pikovsky, A. S. & Rabinovich, M. I. A simple self-sustained generator with stochastic behavior. *Sov. Phys. Doklady* **23**, 183 (1978).

[6] Kuznetsov, A., Kern, M. & Kopell, N. Synchrony in a population of hystersis-based genetic oscillators. *SIAM J. Appl. Math.* **65**, 392-425 (2005).

[7] Koseska, A. & Kurths, J. Topological structures enhance the presence of dynamical regimes in synthetic networks. *Chaos* **20**, 045111 (2010).

[8] Koseska, A., Volkov E. & Kurths, J. Parameter mismatches and oscillation death in coupled oscillators. *Chaos* **20**, 023132 (2010).

[9] Atay, F. M. Total and partial amplitude death in networks of diffusively coupled oscillators. *Physica D* **183**, 1-18 (2003).

[10] Daido, H. & Nakanishi, H. Aging transition and universal scaling in oscillator networks. *Phys. Rev. Lett.* **93**, 104101 (2004).

[11] Tanaka, G., Morino, K., Daido, H. & Aihara, K. Dynamical robustness of coupled heterogeneous oscillators. *Phys. Rev. E* **89**, 052906 (2014).
